# Predicting Known Sentences: Neural Basis of Proverb Reading Using Non-parametric Statistical Testing and Mixed-Effects Models

**DOI:** 10.3389/fnhum.2019.00082

**Published:** 2019-03-18

**Authors:** Bruno Bianchi, Diego E. Shalom, Juan E. Kamienkowski

**Affiliations:** ^1^Laboratorio de Inteligencia Artificial Aplicada, Instituto de Ciencias de la Computación (ICC), CONICET-Universidad de Buenos Aires, Buenos Aires, Argentina; ^2^Departamento de Física, Facultad de Ciencias Exactas y Naturales, Universidad de Buenos Aires, Buenos Aires, Argentina

**Keywords:** electroencephalography, reading, N400, predictability, linear mixed models, cluster-based permutation test

## Abstract

Predictions of future events play an important role in daily activities, such as visual search, listening, or reading. They allow us to plan future actions and to anticipate their outcomes. Reading, a natural, commonly studied behavior, could shed light over the brain processes that underlie those prediction mechanisms. We hypothesized that different mechanisms must lead predictions along common sentences and proverbs. The former ones are more based on semantic and syntactic cues, and the last ones are almost purely based on long-term memory. Here we show that the modulation of the N400 by Cloze-Task Predictability is strongly present in common sentences, but not in proverbs. Moreover, we present a novel combination of linear mixed models to account for multiple variables, and a cluster-based permutation procedure to control for multiple comparisons. Our results suggest that different prediction mechanisms are present during reading.

## 1. Introduction

When performing any task, such as visual searches, listening, or reading, the brain is not passively waiting to be activated by external stimuli. Instead, it is actively trying to predict those upcoming events, planning future actions and anticipating their outcomes (Kveraga et al., 2007). Reading, a natural, commonly studied behavior, could shed light over the brain processes that underlie those prediction mechanisms. In the early 80s, Kutas and Hillyard (1980) observed for the first time a late negative deflection that appeared 400 ms (N400) after the onset of semantically incongruent words compared with congruent words (e.g., “I take coffee with cream and **car**” compared with “I take coffee with cream and **sugar**”). They then described that the amplitude of this N400 was correlated with the *Cloze-Probability* or *Predictability* (i.e., the proportion of subjects that fill in a particular word as the most probable next word in a sentence). They concluded that not only semantic incongruities elicited this negative deflection, but so also did words that had low Predictability (e.g., “I take coffee with cream and **cinnamon**” versus “I take coffee with cream and **sugar**”) (Kutas and Hillyard, 1984).

Two classical views of the N400, the integration interpretation and the lexical-access interpretation, are found in the literature. The integration view proposed that “*it reflects the process of semantic integration of the critical word with the working context”* (Lau et al., 2008). The lexical view suggested that “*it reflects facilitated activation of features of the long-term memory (LTM) representation that is associated with a lexical item”* (Lau et al., 2008). Both views easily explained the gradual nature of the N400: in the former view, because they were more difficult to combine with the previous context and, in the latter, because higher associated word-contexts implied lower difficulty, elicited smaller responses, and was independent of the combination/integration process that updated the context. Some years later, Kutas and Federmeier (2011), proposed a middle-term interpretation, in which the N400 represented the process of binding the current long-term memory (LTM) landscape with the incoming new stimulus. Thus, a large activity was produced when the incoming stimulus mismatched the current landscape. Moreover, the amplitude of this activity was modulated by the degree of mismatch or, inversely, the Predictability of the incoming word (Lau et al., 2008).

Nowadays, most neurolinguistic experiments on predictions use sentences with simple contexts [e.g., “I take coffee (…)”], as in Kutas' first studies. When these statements were presented, it was hypothesized that a subset of words within the semantic field was pre-activated (Lewis et al., 2006) (e.g., “cup”, “sugar”, “toast”, “cream”). But, these “semantic predictions” were not enough for engaging accurate predictions. For instance, in the previous example, the activated words were nouns, but following the rules of English, the statement “I take coffee” cannot continue with another noun. Thus, to generate well-formed sentences, it is necessary to also make “syntactic predictions” (e.g., a preposition like “with”) (Boston et al., 2008). In addition, there are scenarios in natural reading where we find previously known sentences, like in the so called multi-word strings (e.g., idioms, proverbs, song lyrics) (Vespignani et al., 2010; Molinaro et al., 2013). The predictions performed on these sentences are “mnemonic predictions” and, despite the fact that these are found commonly in everyday language, they are largely unexplored in the literature.

The main difference in processing these memory-encoded sentences compared with common sentences is that, in the former, there is a moment where the linguistic context (i.e., the sum of previous words) triggers the recall of the rest of the sentence. Therefore, the upcoming words become highly predictable regardless of whether they are syntactically incorrect or semantically unrelated. That point was called “Recognition Point” (RP: a word that enables the reader to recognize the read sentences) by Vespignani et al. (2010) and “MaxJump” (MJ: a word with the maximal difference in Predictability with the previous word) by Fernández et al. (2014).

To our knowledge, there are very few studies on these memory-encoded sentences, and they are mainly focused in memory-encoded structures within sentences. In several studies, Molinaro et al. explored the first and last words of idioms (e.g., “*break the ice"*) (Molinaro and Carreiras, 2010; Vespignani et al., 2010; Molinaro et al., 2013) and the last word of complex prepositions (i.e., “*in relation to"*) (Molinaro et al., 2008). For instance, in their experiment, they found a larger N400-like component in expected final words compared with unexpected final words (Molinaro et al., 2008), which suggested that Cloze-Probability did not capture all the variables involved in prediction processes. Moreover, two separate late responses were present after the last word of the idiom: a P300 that resembled the expectancy of that word and a N400 sensitive to the semantic properties of that word (Molinaro and Carreiras, 2010). This was further supported by analyzing the first word of the idiom, which generally matched the RP. At that word, semantic violations but not substitutions elicited a N400. In contrast, in the following word, where the context was already known, both elicited a N400 (Vespignani et al., 2010). Moreover, changes in theta and gamma bands and an early increase in fronto-occipital interactions in both frequency bands were observed after the RP and before the final word. That suggested that internal knowledge supported low-level, perceptual processing during reading (Molinaro et al., 2013; Monsalve et al., 2014).

Recently, Fernández et al. (2014) extended the study of multi-word strings to fully memorized sentences, such as proverbs, where they focused on the Predictability effects using eye tracking measures. They found differences in the pattern of fixations between proverbs and common sentences after the MJ (or RP). In accordance with EEG results, these differences were interpreted as a change in the prediction pathways after the recognition of the proverb. This was, to our knowledge, the only study that has explored those sentence types.

In the present work we aimed to find and distinguish brain sources of prediction mechanisms (i.e., semantic, syntactic, mnemonic) in dense EEG signals, when reading different sentence types. With that objective in mind, we focused on analyzing proverb reading and how Cloze-Task Predictability affected word processing, taking into account multiple variables. Since proverbs comes from everyday language, and manipulations would break the memory recall, our corpus consisted in stimuli that were unbalanced in several variables, such as position of the Recognition Point, Predictability, word frequency, and sentence length. Classical hypothesis testing could not cope with these multiple unbalanced co-variables, missing values, or they would require a larger amount of data. To solve these issues, we implemented a Linear Mixed Model (LMM) for each sample (time-point and electrode), which allowed us to test several categorical and continuous co-variables at once. A downside is that this resulted in too many comparisons (i.e., as much as the number of electrodes by time-points). The Cluster-based permutation (CBP) procedure is a non-parametric statistical method that corrects for these multiple comparisons from the sample-by-sample testing in M/EEG data (Oostenveld et al., 2011). This procedure is widely used in the field nowadays, mainly in combination with *t*-test. In this work we replaced the sample-by-sample *t*-test by multivariate LMMs. The combination of these techniques (LMM-CBP) offers a powerful statistical test for M/EEG analysis.

## 2. Materials and Methods

### 2.1. Subjects

Twenty-eight healthy participants took part in the experiment [(24.3 ± 4.2) years old; 12 females], receiving monetary reward for their participation. Three subjects were excluded from analysis due to noisy signal acquisition. Every session took 1.5–2.0 h, which included preparation. All participants provided written informed consent in agreement with the Helsinki declaration, and they were reimbursed monetarily for their participation after the study. All the experiments described in this paper were reviewed and approved by the ethics comittee: “Comité de Ética del Centro de Educación Médica e Investigaciones Clínicas “Norberto Quirno” “(CEMIC)” and qualified by the Department of Health and Human Services (HHS, USA): IRb00001745 - IORG 0001315 (Protocol 435).

### 2.2. Task

**Figure 1:**
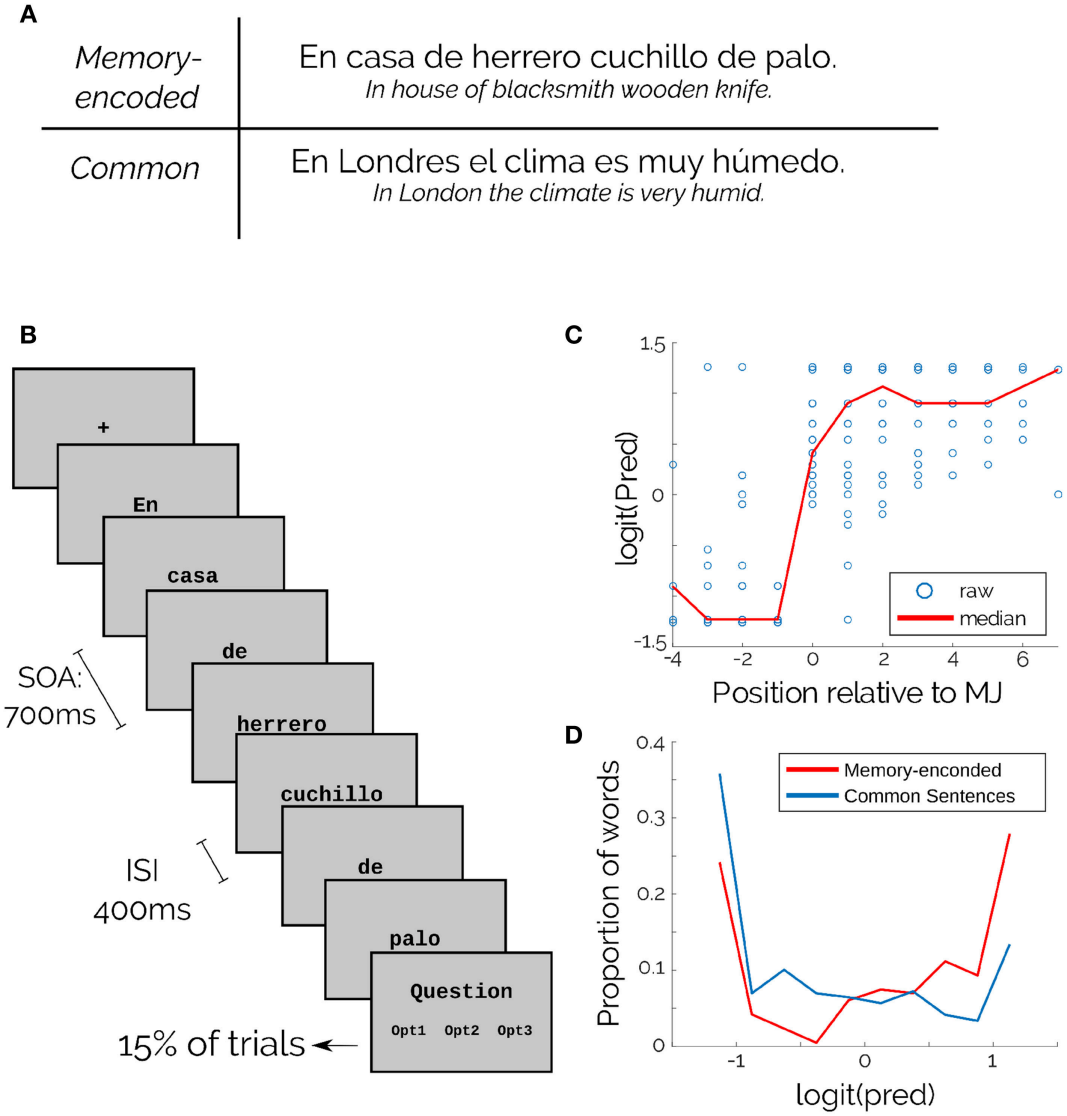
Experimental design and materials. **(A)** Examples of both types of sentences. **(B)** Schematic of the task. **(C)** Word Predictability of memory-encoded sentences. Position is relative to the RP. Blue dots are individual words and red line is the median. **(D)** Predictability of memory-encoded (Red) and common sentences (Blue).

Each trial consisted of an entire sentence (example in Figure 1A) presented word by word in the center of the screen. Every word was presented for 300 ms, with an Inter-Stimulus Interval (ISI) of 400 ms (Figure 1B; SOA = 700ms). The entire trial duration depended on the sentence length (min = 5 words, max = 12 words). Before starting, participants performed 10 trials of training that were not analyzed. Participants were instructed to concentrate on the sentences and to avoid eye movements during trials.

After practice, participants performed 120 trials that were divided into four blocks with unlimited time for the participant to rest between them. A fixation cross lasting 1 s in the center of the screen indicated the start of the trial. Within blocks, the inter-trial interval was 2 s. To ensure that subjects were reading consciously, they answered a simple multiple choice question every six trials (randomized), on average. There was no timeout for answering, and participants were instructed to use this lapse for eye resting. Almost all the responses were correct in all participants (accuracy = 98%).

Visual stimuli were prepared and presented using Psychtoolbox (Brainard, 1997; Pelli, 1997; Kleiner et al., 2007). They were shown on a 19-inch CRT monitor at 60 cm from the participant's eyes at a refresh rate of 60 Hz.

### 2.3. Sentence Corpus

For the present work we selected a subset of 130 sentences from the corpus used by Fernández et al. in a study of eye movements (Fernández et al., 2014). They estimated word-Predictability for every word, using a Cloze-Task (Taylor, 1953), with 18 graduate and undergraduate students. In this task, subjects had to fill the most probable word following an incomplete sentence. Word-Predictability was then estimated for each word as the proportion of correct answers. Our subset included 50 memory-encoded and 80 common sentences, and 5 sentences of each category were used in the training stage. For the memory-encoded sentences, it was possible to find a Recognition Point or Max Jump (Fernández et al., 2014) for each sentence (Figure 1C).

The corpus consisted of 897 words (470 content words) with 451 unique words (368 unique content words). The median logit Predictability for memory-encoded sentences was 0.186 ± 0.948, and for common sentences was -0.430 ± 0.897 (Figure 1D).

### 2.4. EEG Recording and Preprocessing

Electroencephalography (EEG) signals were recorded using a Biosemi Active-Two (Amsterdam, The Netherlands) 128-channel system at 1024 Hz. All the analyses were performed using EEGLAB (Delorme and Makeig, 2004; Makeig et al., 2004), FieldTrip (Oostenveld et al., 2011), and in-house MATLAB and R scripts. Data were re-referenced to linked mastoid. A Hamming-windowed FIR band-pass filter of 0.1–40 Hz was applied, using “eegfiltnew” in EEGLAB v14.1 (Widmann and Schröger, 2012), and data were downsampled to 256 Hz. For the Event-Related Potential (ERP) analysis, data were epoched from 100 ms before to 700 ms after the onset of the stimulus, with the amplitudes from -100 ms to the onset as the epoch baseline. Epochs with >5 electrodes with at least one sample of 80 μV were rejected. Ocular artifacts were detected using Independent Component Analysis (ICA) and removed after manual inspection of the components for typical ocular topography.

### 2.5. Analysis and Statistics

We implemented four types of analyses (Table 1), which are described in detail below. Briefly, some approaches use *a priori* defined ROIs and time-window to extract a single value for each trial. Then, it is possible to discretize variables, average across categories, and apply different hypothesis tests –such as Kruskal-Wallis or Wilcoxon's tests–, or to preserve the continuous variables and apply a regression analysis. Nevertheless, these approaches imply a huge loss of information in the averaging procedure. In order to avoid that, it is possible to run a test in each sample (electrode and time-point) and deal with the multiple comparisons problem using for instance a cluster-based permutation (CBP) procedure. This procedure is widely used in multichannel recordings, since it takes into account the high correlations between channels with little loss of power, as opposed to Bonferroni or false-discovery rate approaches (Maris and Oostenveld, 2007).

**Table 1:** Statistical approaches on analyzing EEG data for the Predictability effect (or any continuous variable).

	**N400 window**	**Complete epoch**
Discrete Predictability	Kruskal-Wallis and Wilcoxon	CBPT (*t*-test)
Continuous Predictability	Regression	LMM-CBPT (NEW)

#### 2.5.1. Categorical Predictability in the N400-window

**Figure 2:**
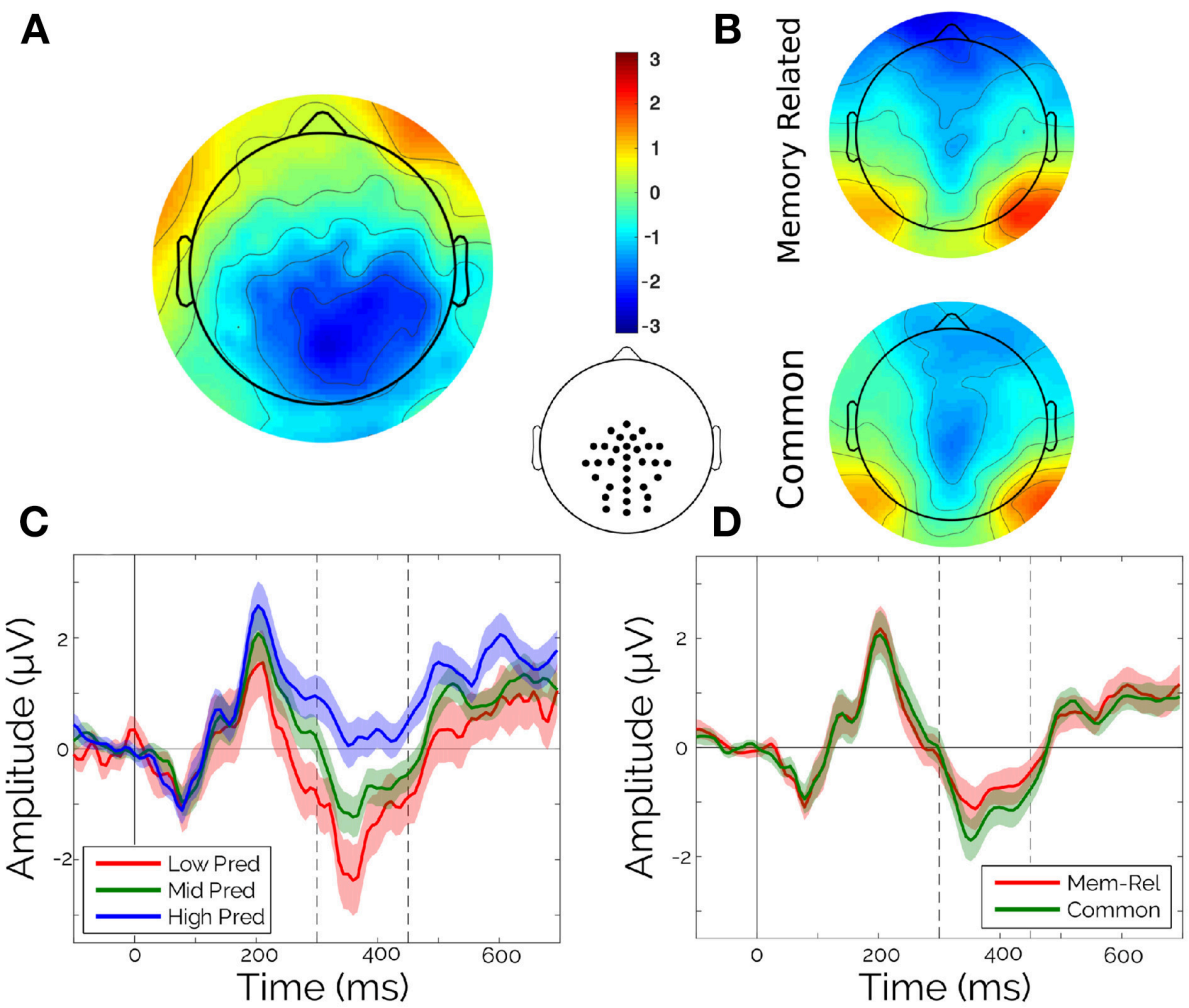
Classical ERP analysis. **(A)** Scalp topography of the difference between High and Low terciles of Predictability in the [300 450]ṁs interval. **(B)** Scalp topography for memory-encoded (top) and common (bottom) sentences, in the [300 450]ṁs interval. **(C)** Average waveforms for a centro-parietal cluster (see inset) separated by terciles of Predictability. **(D)** Average waveforms for a centro-parietal cluster (see inset) separated by sentence type. Vertical lines indicate the [300 450]ṁs interval.

Only content words (adjectives, verbs, and nouns) were kept for all the analyses. Words shorter than three characters, and the first word of each sentence were also rejected. All the words were classified independently in terciles according to the values of both Frequency and Predictability of the corpus. Evoked potentials were averaged in the chosen ROI (Figure 2, central inset), time-window (between 300 and 450 ms), condition (tercile of Frequency or Predictability, and Sentence Type) and participant.

The average for each participant in the N400-window were submitted to a Paired Wilcoxon Rank-Sum Test with Sentence Type (two levels) as main factor, and to two independent Kruskal-Wallis Tests with Predictability (three levels) and Frequency (three levels).

#### 2.5.2. Continuous Predictability in the N400-window

A Linear Regression was performed to address for the continuous Predictability effect (using its raw numerical value) on the N400-window. The Linear Regression was fitted using single-trial values from all the participants together, with participants included as dummy variables.

#### 2.5.3. Categorical Predictability of the Whole Epoch

In order to detect predictability effects occurring on the whole epoch –i.e., on any time point or electrode–, a common solution is to apply a CBP test, in which averaging across electrodes and time points is not required. The current Fieldtrip's implementation supports mainly categorical factors (Maris and Oostenveld, 2007; Oostenveld et al., 2011). This toolbox was used to run two separate tests for Predictability (two levels: High vs Low) and Sentences Type (two levels).

#### 2.5.4. Continuous Predictability of the Whole Epoch

The use of any type of natural sentence that appears in everyday vocabulary implies using a non-uniform corpus of sentences; frequency, length, Predictability, and other word properties cannot be controlled and balanced across trials. This is an important issue when using classical ERP analysis, because when averaging across one condition other conditions may become unbalanced. In recent years, computational advances have allowed researchers in the neurolinguistics field to handle this issue using linear regressions, both in the study of eye movements and in ERP analysis. In the former case, Linear Mixed Models (LMM) have become the most common methodological technique, because they allow for testing multiple co-variables at once, and they also account for random effects (e.g., of subjects and items) that are necessary to avoid the language-as-fixed-effect fallacy in studies that involve natural language (Clark, 1973). LMMs are not widespread in the ERP field. To our knowledge, the only implementation of LMM applied to ERPs is in the LIMO toolbox for MATLAB (Pernet et al., 2011), but it is focused on “assessing the inter-subject variability” and not for testing for effects of co-variates. Finally, other classical regression analysis have just been introduced in the last few years (Hauk et al., 2006; Smith and Kutas, 2015a,b), but without the benefits of LMMs and CBP pocedure stated above.

After estimating the effects for each co-variable, another important benefit of fitting LMMs is that it is possible to extract predictions (partial effects) from the original data. The *remef()* package for R (Hohenstein and Kliegl, 2013) takes the results of a LMM as input and uses them to remove the partial effects for those co-variables from the original data. Analyzing this newly generated data allows us to understand further the remaining cleaner effects.

In the present work, LMMs were fitted using lme4 package V1.1-12 (Bates et al., 2015b) for R V3.3.2 (R Development Core Team, 2008) as follows: each time (*t*) and electrode (*e*) sample of all the epochs were used as dependent variables. A combination of co-variables (and some interactions) were used as independent variables in the following model:

(1) $$\begin{array}{l} Amp_{\left(t,e\right)}\sim Freq+Pred:Type+Pos:Type+(1|Subj)\\ \;+(1|Word) \end{array}$$

where *Freq* is the Frequency on the lexicon, *Pred* is the Predictability, *Type* refers to the Sentences Type, and *Pos* is the Ordinal Position in the sentences. Subject ID (*Subj*) and the string of each word (*Word*) were used as random factors. The colon between two co-variables indicates that we are testing the interaction between them. The relevant output of these models is an estimate of the slope and its error (SD) for each of the fixed factors in Equation 1. With this information it is possible to calculate a *t*-value, which represents how far away from zero the estimate is. Commonly, if the estimate is more than 2.0 SD away from zero, the slope is considered significant with α < 0.05 (Fernández et al., 2014).

Because this model was fitted for each electrode-time sample, the final results of the analysis were *n*-by-*m* *t*-values matrices (i.e., one matrix with the *t*-values of each model run for each term of the model), with *n* electrodes (128) and *m* time samples (103). This means that more than 10,000 *t*-values were in consideration at once, which is a huge number of comparisons that needs to be corrected for multiple comparisons to control for Type I error. To solve this multiple comparison issue without losing statistical power, we implemented a CBP protocol proposed by Maris and Oostenveld (Maris and Oostenveld, 2007), and we adapted it to the LMMs.

##### 2.5.4.1. Permutation procedure

Maris & Oostenveld (Maris and Oostenveld, 2007) introduced the CBP procedure that proposed a novel way to analyze EEG data in a non-parametric framework, which avoided *a priori* hypotheses of time and scalp distribution. This procedure consists of running a statistical test for each electrode-time sample.

The CBP procedure includes the following steps:

1. Perform a statistical test for every electrode-time sample amplitude.
2. Select all samples that have a *t*-value larger than some *t*_*th*_ threshold.
3. Cluster the selected samples in connected sets on the basis of temporal and scalp distribution adjacency (at least two neighbor samples, electrodes and/or timepoints).
4. Calculate a cluster-level statistics (e.g., by taking the sum of the *t*-values within a cluster).

To define a significant cluster, a permutation procedure was used. The labels of categories of the trials were shuffled randomly and the previous procedure was repeated (steps 1–4) for each permutation *p*. Then, the largest cluster of each permutation was selected, and all the *t*-values within this cluster were summed (*MaxSum*_*p*_). The *MaxSum*_*p*_ values of many permutations (in our case *N*_*P*_ = 500) were collected to build a distribution. The sizes of the original clusters (*t*_*cluster*_) were compared to this distribution of *MaxSum*_*p*_. Then the p-values for each original cluster were estimated as the proportion of *MaxSum*_*p*_ that exceeds *t*_*cluster*_, over the whole set (*P*) (Equation 2). In the case that none of the permuted datasets exceeds the original data, the p-value is defined as less than 1 over *N*_*P*_ (Equation 2).

(2) $$\{\begin{array}{l} if\sum_{p\in P}(MaxSum_{p}>t_{cluster})>0,\;p=\sum^{\text{​}}p\in P\\ \;(MaxSum_{p}>t_{cluster})/N_{P}\\ if\sum_{p\in P}(MaxSum_{p}>t_{cluster})>0,\;p<1/N_{P} \end{array}$$

Finally, an alpha level is defined to determine the significance level of these clusters as in parametric testing.

In the case of a *t*-test, a single *t*-value is obtained from each test. Hence, a single distribution of cluster sizes (sum of *t-*values) was built from the permutations, and the size of the original clusters wwere compared to this distribution. In the case of LMMs statistics, one *t*-value was obtained for each fixed effect included in the model. Each of these values was treated separately. A single distribution of cluster sizes was obtained for each fixed effect, and the sizes of the clusters of the original models were compared with these distributions.

The main problem faced in the adaptation of a CBP protocol to LMM statistics was the multiple co-variables that need to be considered at the same time. In a multivariate analysis, where there are correlations between the co-variables, shuffling only one label would break the correlations between all the covariates. To avoid this, the trial label was shuffled, and the entire structure of correlations was keep intact. That is, each EEG matrix (i.e., the matrix with the EEG amplitudes) was assigned to a new co-variable vector (i.e., the vector with all the co-variables from a trial).

Another important problem to solve in the permutation of trials under a LMM is that permuting across the random variables breaks the random factor structure, wich generates anti-conservative results. To address this issue, we mimicked the F1/F2 approach used in psycholinguistics when fitting ANOVA models for more than one random variable (i.e., words and subjects). Here, we permuted within each of the random factors (Figure S1). That is, for our model, which had two random factors (i.e., subject and word), we first ran a complete CBP procedure that kept the structure for Subject and, second, we ran it again keeping the structure for Word. In the results we present both of these analyses.

##### 2.5.4.2. Implementation

Because the major literature in LMM is based on the lme4 library for R, to implement this CBP procedure it was necessary to export data from MATLAB structures to CSV files. To facilitate the parallelization, data were exported in many CSV files, one per time sample, with all the information on amplitudes, co-variables, and random effects for each electrode. The in-lab parallelization was made using 26 4-core, *Core i7* Desktop computers (104 independent cores) at the same time to fit all the models. It took 6 min to fit all the permutations for one electrode and one time sample (*N* = 500), which made it possible to run all the models (i.e., 128 electrodes in 103 time-samples) in 12 h. For model fitting, RAM memory usage was negligible in relation to the processing cost. Additionally, it is important to remark that for each permutation, all the electrode-time samples received the same shuffling, which was pre-calculated and stored in each core of the 26 computers. The code for this analysis is available at http://reading.liaa.dc.uba.ar. It includes scripts written in MATLAB, R, and Bash, example data, and a tutorial.

## 3. Results and Discussion

### 3.1. Predictability and Sentence Type Effects: Classical Approaches

As a first step, we aimed to assess the main effects of Word Predictability, Sentence Type, and Frequency in the evoked responses. Particularly, guided by the literature on Predictability effects, we looked for Predictability effects in the N400 window ([300 ms, 450 ms]; and selected electrodes, see Figure 2, central inset). Within this N400 window, only Predictability showed a significant effect (Figures 2A,C, Kruskal-Wallis: χ^2^ = 9.02, *p* = 0.011). Frequency (Figure not shown, Kruskal-Wallis: χ^2^ = 1.38, *p* = 0.50) and Sentence Type (Wilcoxon: *z* = 1.69, *p* = 0.091) showed no significant effects (Figures 2B,D).

However, it was possible to identify two main limitations of this type of categorical ERP analysis: on one side, for the Predictability Effect it is necessary to rely on a categorization of this continuous variable. On the other side, averaging across electrodes and time samples implies introducing *a priori* hypotheses about the effect distribution. This is critical when expecting potential effects from other co-variables. For instance, the N400 window was mainly motivated by the Predictability effect, but Frequency and Sentence Type, as well as Predictability itself, could, in principle, have effects outside this window. To overcome the first limitation, a linear regression was fitted for the numerical (logit) Predictability against the mean amplitude on the N400 window, with Subjects as a dummy variable. Extending the previous analysis, we observed significant effect of Predictability on the N400, but the variance explained by this factor was very low (β = 1.1567, *R*^2^ = 0.0144, *p* < 0.01; Figure S2A).

In order to avoid averaging across electrodes and time points, and to overcome the second limitation, a non-parametrical cluster-based analysis was performed for both the categorical Predictability (two levels, High vs Low) and the Sentences Type. Predictability effect (Figure S2B) showed one significant cluster (*p* = 0.002), while the Sentences Type did not show any significant effect. The significant cluster of Predictability appeared approximately at the expected latency and location, i.e., between 300 and 450 ms over the centro-parietal electrodes. However, the present analysis enabled us to explore the dynamics of significant electrodes in more detail, in particular it was a little bit earlier (210 to 420 ms) than the a priori defined window, covering the maximum number of electrodes at 335 ms (Figure S2C).

### 3.2. Predictability, Sentence Type and Position Effects: LMM-CBP Approach

In the previous subsection we introduced two alternative analyses that independently solved the limitations found when analyzing the Predictability Effect. On one side, regressions allowed to use continuous variables. On the other side, CBP procedure with *t*-test allowed to avoid making *a priori* hypothesis about latency and scalp distribution, which was particularly relevant when analyzing unexplored effects, like Sentences Type. In the following, we combined Linear Mixed Models (LMMs) with a CBP procedure (LMM-CBP), in order to solve all these limitations in a single procedure.

**Figure 3:**
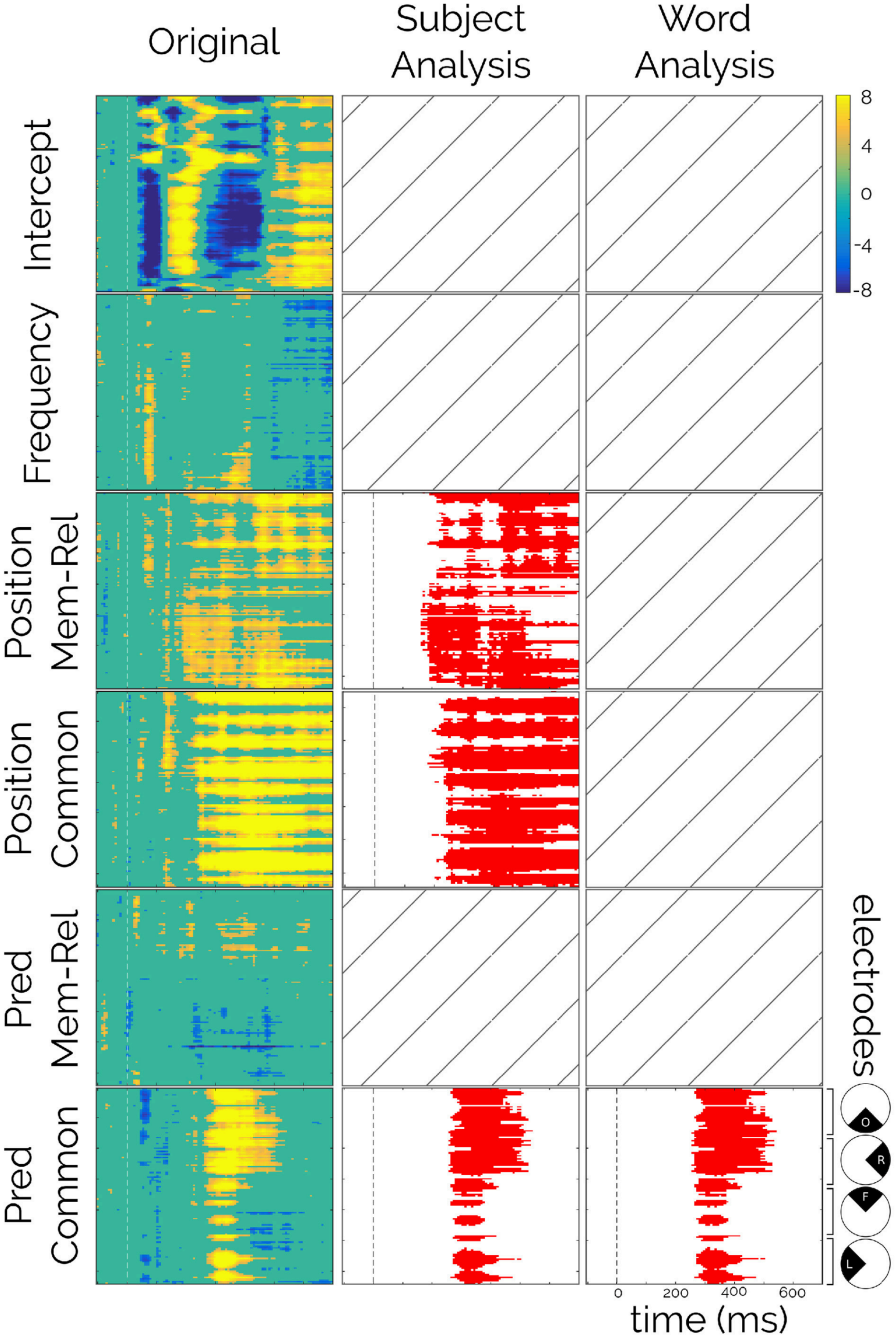
Linear Mixed Models results. *t*-values for each fixed factor (left column), significant positive *t*-values (>2) are presented in orange-yellow colors, significant negative *t*-values (< −2) are presented in blue colors, and non-significant *t*-values are presented in green. Significant clusters after CBP procedure for subject analysis (center column) and for word analysis (right column). Crossed squares represent no significant clusters for that effect after the correction. Rows correspond to the fixed effects: Intercept, Frequency, Position × Mem-Rel (Memory-related), Position × Common (Common words), Pred (Predictability) × Mem-Rel (Memory-related), and Pred (Predictability) × Common (Common words). Columns correspond to *t*-values from the LMM (original), and the results from Cluster-Based Permutation procedure using Subjects or Words.

The first step in the LMM-CBP procedure was to run the statistical model (Equation 1) for each time-electrode sample. Each model was fitted with 9,459 epochs of 25 participants. The results were summarized in one matrix for each fixed effect of the model (Figure 3, first column). These matrices showed only the significant *t*-values before the multiple comparison correction (i.e., in blue, *t* < −2; in yellow *t*>2).

As explained in the Methods Section, the permutation of the labels was performed in two parts. First, we kept the subject structure intact (Figure S1B and Figure 3, second column) and second, we kept the word structure intact (Figure S1C and Figure 3, third column).

First, the intercept term, that is, the ERP amplitude when all the co-variables equals their own mean, resembled the usual evoked responses to visual stimuli (Figure S3, an early negativity N1, followed by a positivity P2, etc.) irrespective of their frequency, Predictability, position, and context. This result was expected since the Intercept should equal the mean accross all conditions (Smith and Kutas, 2015a), and it allowed us to highlight the power of this novel method to capture relevant effects in ERP signals (Figure 3, first row).

**Figure 4:**
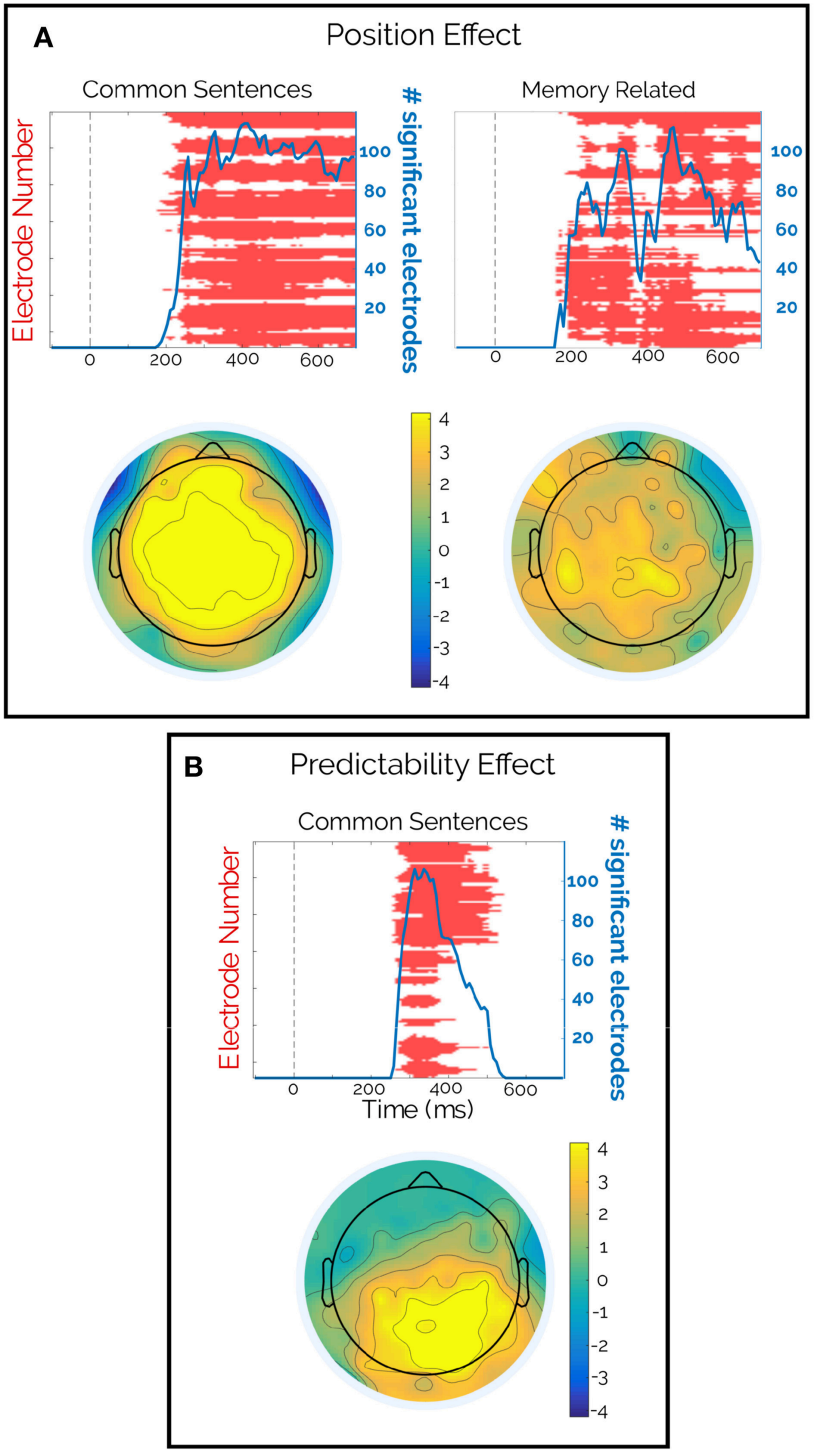
Scalp and time distributions of significant effects. **(A)** Significant clusters (red) and cluster size (blue) as function of time (top), and topographical distribution of the *t*-values in the [300 500]ṁs interval (bottom) for the Position effect for both sentence types. **(B)** Significant clusters (red) and cluster size (blue) as function of time (top), and topographical distribution of the *t*-values in the same [300 500]ṁs interval (bottom) for the Predictability effect in common sentences. Only the subject permutation scheme is presented, because the Word permutation did not have significant clusters for the Position effect, and it had a very similar cluster in the Predictability effect.

Second, a significant effect of the word position was observed for both types of sentences that was only significant for the subject permutation scheme (Figure 3, third and fourth rows, *p* < 0.002). This spatially widespread effect showed a latency of 170 ms (memory-encoded: 165 ms and common: 180 ms) and lasted the entire epoch (Figure 4A). Moreover, this effect seemed to be present regardless of the Predictability effect (Figure 4B). We attribute the absence of this effect in the word permutation scheme to the composition of the sentence corpus, where each word was presented only a few times across sentences (mean [range] = 2 [1 12]), and most of them appeared only once in the corpus (345 out of 448). Thus, when permuting within words, the ERP space-time matrix of a given word and a given subject was assigned to the same word of another subject (only changing the subject ID). This led to a very conservative criterion, that only significant effects, like the Predictability on common sentences (see below), were able to attain.

Finally, the Predictability analysis presented a significant cluster, with both permutation schemes (Figure 3, bottom row, *p* < 0.002 and *p* = 0.005 respectively) between 258 and 540 ms. Although widely spread across the scalp, this effect was stronger in the centro-parietal region and resembled the distribution of the N400 (Figure 4B). Importantly, this effect was only seen for common sentences and not for memory-encoded ones (Figure 3, fifth row).

**Figure 5:**
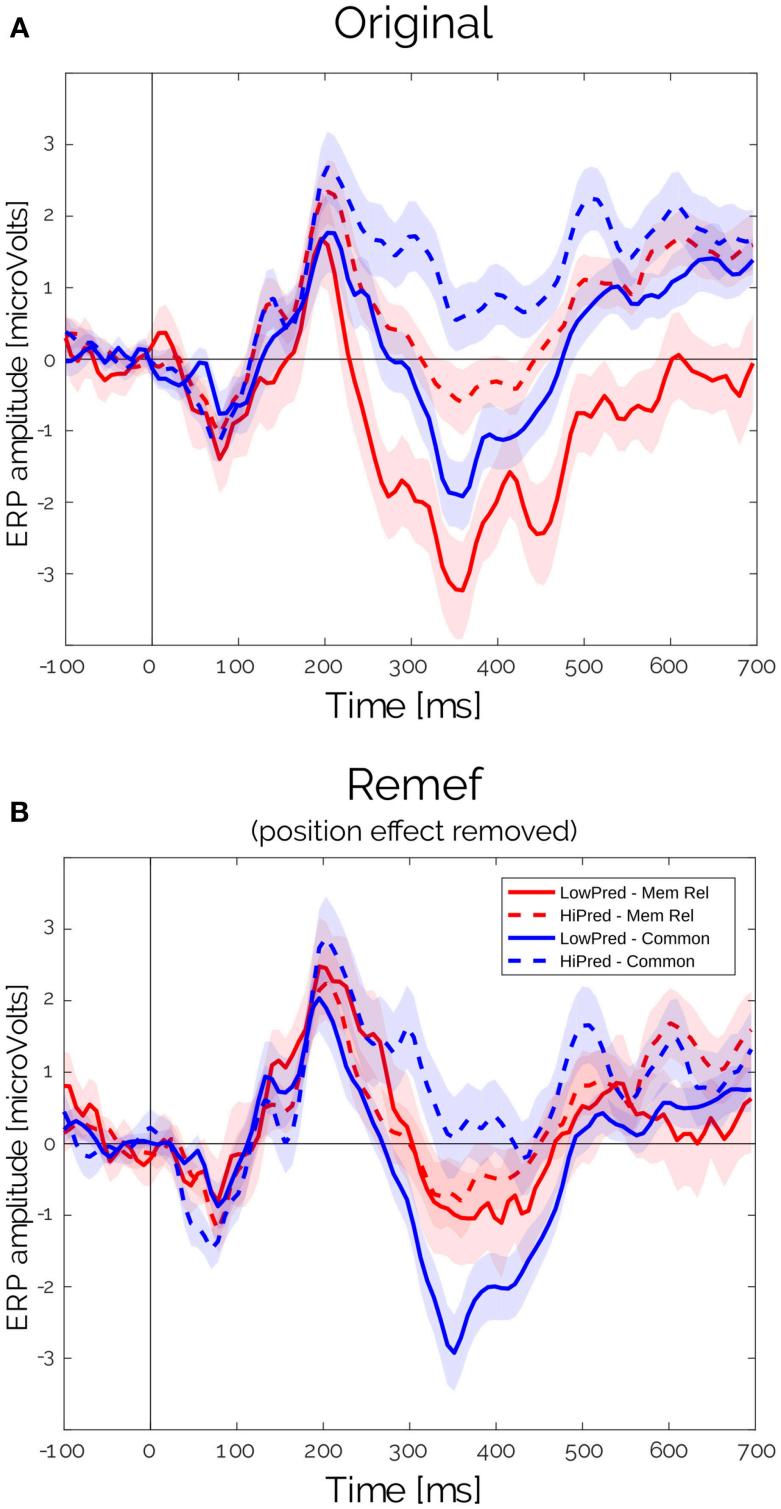
ERP for Predictability × Sentences type for **(A)** original EEG data and **(B)** after removing the predicted position effect, as obtained from CBP procedure.

The LMM analysis allowed us to make a distinction between the contributions of different co-variables. Furthermore, it makes possible to remove some of those partial effects that acted as confounding factors for the effect of interest. Initially, the huge Position effect overlapped the Predictability effect (Figure 5A). But, after estimating the partial effects, we were able to isolate and remove the effects of Position for both sentence types and study clean waveforms for the Predictability and Sentence Type interactions. As expected from the results from Figure 3, we observed a clear difference between High and Low predictable words for the common but not for the memory-encoded sentences in the N400 time-window (Figures 3, 5B). Interestingly, the N400 amplitude for memory-encoded sentences was closer to the High predictable than to the Low predictable words (Figure 5B), suggesting the absence of N400 for either high and low predictable words in the memory-encoded sentences.

## 4. Conclusions

The aim of the present work was to investigate the electrophysiological basis of different prediction sources in reading. To achieve this, we studied mnemonic (i.e., recalling a sequence of words from long term memory), semantic (i.e., based on the sentence topic), and syntactic predictions (i.e., predictions on syntactical rules). We used a variety of memory-encoded sentences from popular culture, like proverbs, song titles, or parts of song lyrics. All these sentences have a recognition point (RP). By using this type of natural stimuli we faced an important imbalance in the variables, such as the position of the RP, the Predictability and sentence length. This imbalance generates several difficulties for some ERP analyses commonly used, in which variables are studied one at a time, averaging across all the residual variables. In addition, these analyses require variables to be categorical –loosing information in the case of continuous variables–, and to collapse the EEG time samples and electrodes in a single value. To overcome these limitations, two partial solutions are usually applied: a linear regression, that allows modeling with continuous variables; and the Cluster-Based Permutation (CBP) procedure with *t*-test as the sample-by-sample test (Maris and Oostenveld, 2007), that allows studying brain potentials without any *a priori* hypothesis on the latency and the spatial location. Nevertheless, despite the results we obtained from these analyses were in line with the N400 bibliography, they were not a conclusive solution for those limitations, as they couldn't tackle all the limitations at once.

Taking into account those considerations, we developed a novel analytical approach. In the rERP framework proposed by Smith and Kutas (2015a), they used multivariate regressions for each sample of the epoch matrix to separate the spatial and temporal dynamics of each effect. They ended up with one time series of beta values for each effect, which was the core of their proposal. But, after that, they analyzed betas as a typical –but cleaner– ERPs (Smith and Kutas, 2015b). Here, we showed that is possible to use Linear Mixed Models (LMM) rather than regressions to generate better modeling of the data (Baayen, 2008; Bates et al., 2015a,b). And, instead of going back to the classical statistical approaches, we take advantage of the powerful statistics generated by the LMMs for each sample, and combined them with the CBP procedure introduced by Maris and Oostenveld (Maris and Oostenveld, 2007). This procedure enabled us to analyze the significance of the model slopes with a non-parametrical test for solving the multiple comparison issue generated when comparing many statistical results.

Two additional challenges were solved for our implementation: (1) the processing cost; and (2) the decision on how to perform the flag permutation of trials, as the trials could be grouped by subjects or words. The former was worked out by parallelizing the analysis. We run this in a cluster but it also could be done in a single multi-core computer. And the last one was solved by permuting in two stages –i.e., performing “subject” and “word” analysis separately–, based on the F1/F2 analysis that was traditionally used in linguistics before the popularization of LMM.

Interestingly, the proposed LMM-CBP procedure was able to model the raw ERP in the intercept term, tearing apart all the modeled effects. This is expected from the mathematical derivation of the rERP framework done by Smith and Kutas and, although it doesn't add new information, it serves as clear validation of the method (Smith and Kutas, 2015a,b). Furthermore, based on similar ideas, we were able to separate the effects of Predictability and Word Position in Sentences, which overlap on latency and scalp distribution.

The main result of this non-parametric analysis was a clear and significant effect of the word Predictability on the N400 time window, but only for the common sentences in both permutation scenarios of subjects and words. The N400 effect strongly arose without using *a priori* hypothesis of latency or localization, and it was not present in memory-encoded sentences. Interestingly, this suggests that there was neither facilitation nor a combinatorial process that relied on the previous context. This could be the case if proverbs were actually loaded from memory as a whole construct that is recalled after the recognition point was read. Moreover, the activity during the N400 period at the same centro-parietal cluster of electrodes was smaller for memory-encoded than for common sentences, as it was shown when removing the Position effect. This is in line with the results of Molinaro et al. who observed a larger negative activity in the N400 time window for different substitutions compared with original idioms or collocations (Molinaro and Carreiras, 2010). Importantly, instead of comparing between sentence types, in our case, we evaluated the effect of Predictability within each type, and no substitutions were used. Our effects, although smaller, allowed us to use the gradual nature of the N400 as a hallmark. These results suggested that the N400 was not only smaller in proverbs, but it was also insensitive to Predictability.

In addition to this N400-like effect, we observed a clear position effect that was spread widely in the scalp, which started after 200 ms of word onset. This positive drift in the EEG signal as the reader moved through the sentence was present for both sentence types. This effect could be related to a cumulative integration process. Future approaches need to parametrize information along the sentence to be included in LMMs and to relate these effects to a specific cognitive model (de Lange et al., 2010; Brouwer and Hoeks, 2013; Kamienkowski et al., 2018).

It is important to note that the position effect was only significant in the subject analysis. This could be due to the fact that when permutations were generated for the subject analysis, all the epochs of each subject were permuted within the same subject. Because we had 354±76 valid epochs (after filters, see methods) per subject, we linked each ERP matrix to a different word, but, always from the same subject. Thus, the new “random” analysis broke the word structure, which generated random results for this item. Conversely, in the word analysis the permutation was done across the words (using the unique strings as the “word” levels). This means that if a word appeared only once in the entire corpus (which most of the words did), permutations were assigned an ERP matrix from one word of one subject to the linguistic information of that same word, but from another subject with a probability of ~96%. This resulted in similar results in the permutation analysis as in the original analysis and to a high MaxSum statistic for the cluster selection, rejecting all the clusters in the original data. Thus, it was not possible to draw reliable conclusions from the Word Analysis.

Nevertheless, beyond the methodological discussion, the position effect in the subject analysis suggests a potential cumulative process during sentence reading. This effect could be separated from the cloze-task predictability effect with the present approach. In order to further analyze the slight differences in scalp distribution and strength of the position effect a follow up study could be designed using longer sentences, aligned by their Recognition Point. This would decrease the number of confounding effects.

In the present work, we present a novel analysis by combining Linear-Mixed Models and a cluster-based permutation procedure. The former are becoming very popular in eye movement and reading studies to cope with multiple, continuous, independent variables. The latter is very popular in EEG analysis and it is used to deal with the usual multiple-comparisons problem in the high density EEG signal. Using the novel LMM-CBP technique, we showed that different mechanisms are involved in the prediction of forthcoming words. Future experiments should investigate these mechanisms further to describe the precise brain areas involved and the contributions of timing and frequency, to then integrate them with cognitive models of the role of prediction in processing natural language.

## Data Availability

The datasets analyzed for this study can be found in https://github.com/brunobian/NeuralBasesProverbReading2018 and in http://reading.liaa.dc.uba.ar. More detailed or complementary data are available on request.

## Author Contributions

BB, DS, and JK designed the study and wrote the manuscript. BB collected and analyzed the data. BB and JK discussed and interpreted the results.

### Conflict of Interest Statement

The authors declare that the research was conducted in the absence of any commercial or financial relationships that could be construed as a potential conflict of interest.
