# Supplementary material for: Predicting Known Sentences: Neural Basis of Proverb Reading Using Non-parametric Statistical Testing and Mixed-Effects Models
Source: Front Hum Neurosci. 2019 Mar 18;13:82. doi: 10.3389/fnhum.2019.00082 (PMC6434989; doi:10.3389/fnhum.2019.00082)
Supplement: Supplementary file 1 [file Data_Sheet_1.PDF]

Supplementary Material: Predicting known sentences: neural basis of proverb reading using nonparametric statistical testing and mixed-effects models.

1 SUPPLEMENTARY FIGURES

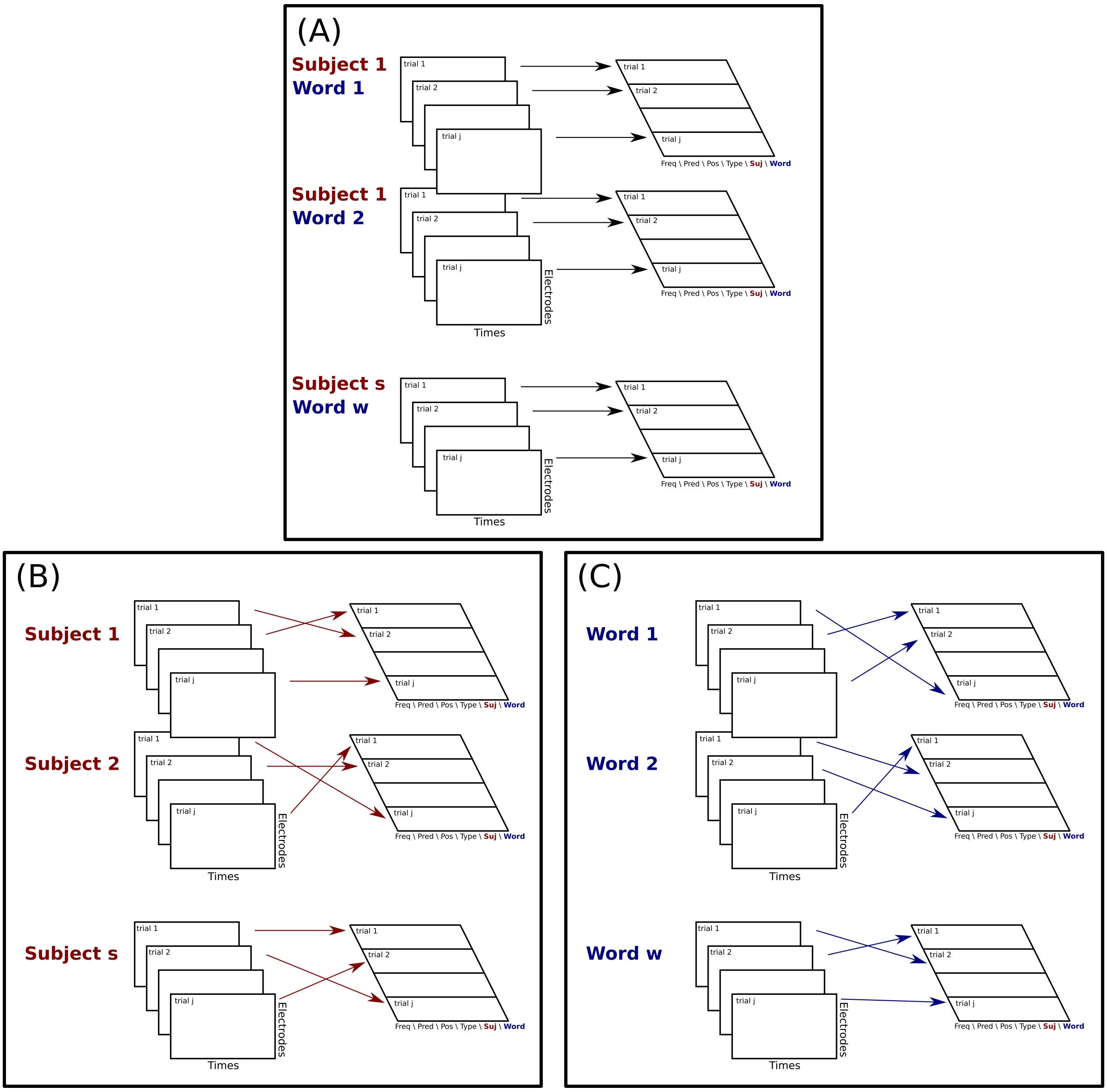

Figure S1. Permutation design. (A) Original data. Each subject and word has associated a 2D matrix with amplitudes for each time and electrode, and each of these matrices is linked with an unique vector with the features of that word (frequency, predictability, length, sentence type, etc). During the permutation procedure, the 2D matrices are re-assigned to different feature vectors, changing the subject label (B) or the word label (C), but preserving the structure of spatial and temporal correlations on one side, and within feature vectors on the other side.

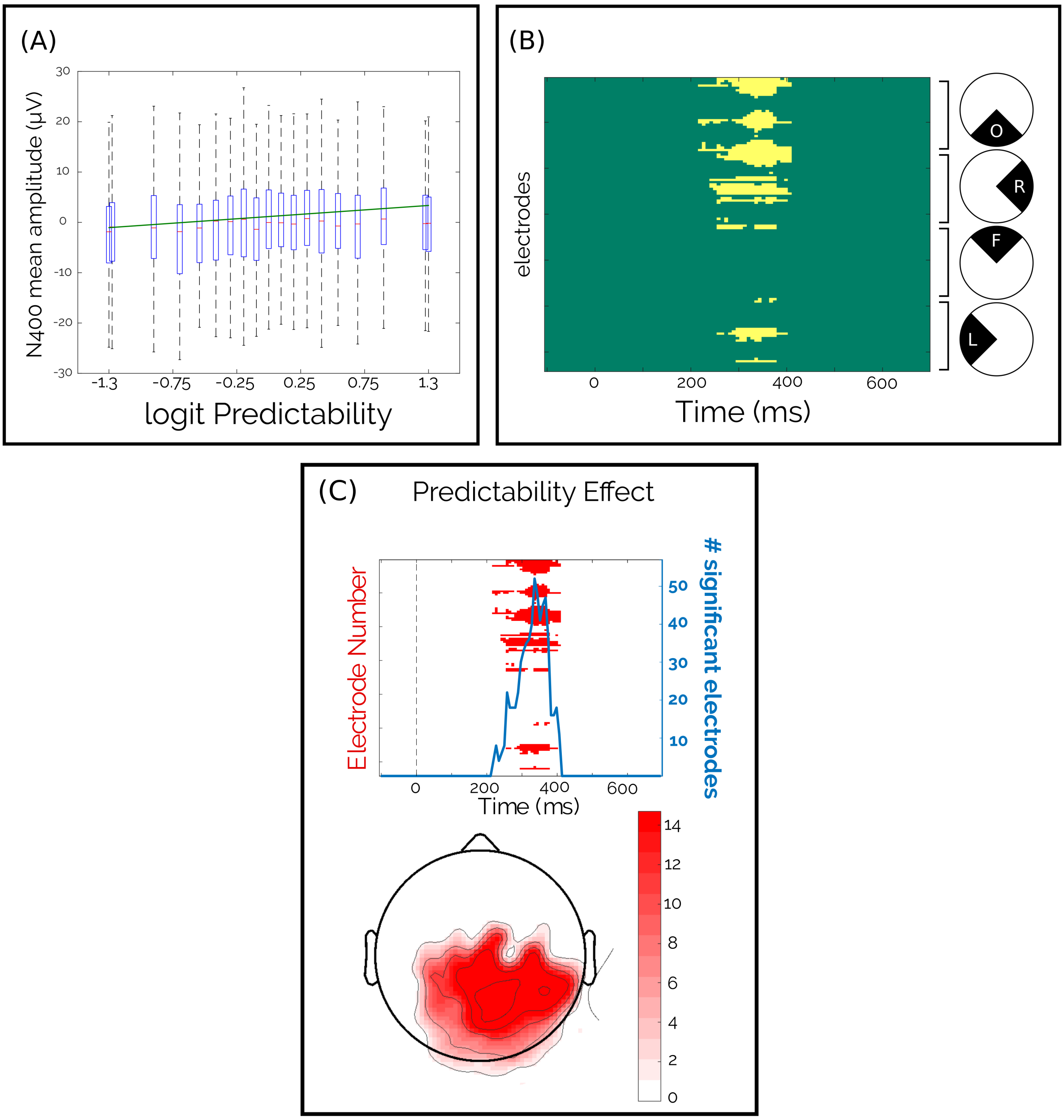

Figure S2. Predictability analyses. (A) Linear Regression of trail Predictability on mean amplitude of N400 ROI between 300 and 450ms. (B) t-test Cluster Based Permutation Test for High and Low Predictability. Significant cluster (negative) p $= 0.002$. (C) Scalp and time distributions of predictability significant cluster. For the scalp distribution, the sum of significant samples between 300 and 450ms.

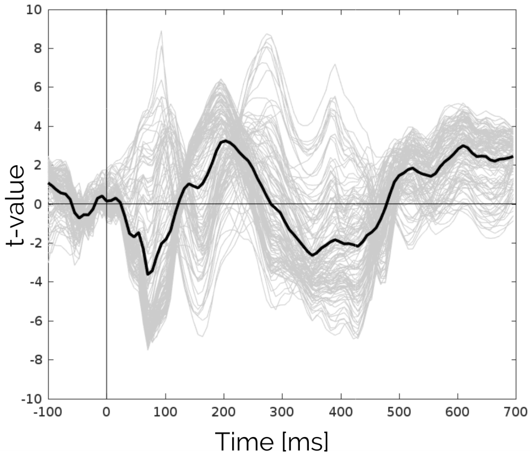

Figure S3. Intercept partial effect. t-values for all the electrodes (gray) and the average (black) from the fitted model.
